# Nuclein in Tuberculosis

**Published:** 1910-03

**Authors:** James Alexander

**Affiliations:** Bennett Medical College, Chicago; 1360 Fulton St., Chicago, Ill.


THE
### TEXAS MEDICAL JOURNAL.
Established July, 1885

F. E. DANIEL, M. D., - - - - - - Editor, Publisher and Proprietor

Published Monthly.—Subscription $1.00 a Year.

VOL. XXV AUSTIN, MARCH, 1910. No. 9

The publisher is not responsible for the views of the contributors.

### Original Articles.

For Texas Medical Journal.

### Nuclein in Tuberculosis.

BY JAMES ALEXANDER, M. D., BENNETT MEDICAL COLLEGE, CHICAGO.

The other evening I had the pleasure of attending a meeting of the Chicago Academy of Medicine, and listening to an extremely important and interesting paper by Professor Ward, of St. Louis. In this paper Dr. Ward described his new method of treating tuberculosis. This consists in the intravenous injection of nuclein, diluted with normal salt solution. The dose of nuclein solution given was twenty minims, gradually raised to one dram. The injections were repeated every one to seven days, according to the degree of febrile reaction following. This was greater in cases of mixed infection, with strepto- and staphylococci; but the benefit seemed most decided in purely tubercular infection.

Dr. Ward presented a tabular statement detailing the results in fifteen out of about fifty cases, on which his report was based. He had been working on this matter nearly three years. He began by an extended study of the blood of tubercular patients, in which he discovered that the specific gravity of such blood is below normal, the hemoglobin is deficient, the erythrocytes diminished in number and the proportion of degenerate or imperfect forms very large. During the course of his experiments the blood was examined every week; and in the fifteen cases presented the period of twenty-four weeks was covered.

The report showed that the nuclein injections were followed by a steady improvement in the condition of the blood, the specific gravity rising to normal, the hemoglobin increasing and the red corpuscles of imperfect form becoming fewer. At the same time the number of tubercle bacilli in the sputa became fewer until they finally disappeared. Of the fifteen cases nine were cured, three improved and three died. Most of the cases were well advanced, they being selected as the least promising of all those under Professor Ward's treatment. In the fatal cases there were personal circumstances interfering with the persistent carrying out of the treatment, which may have had much or little to do with its failure.

Taken altogether, the report was a remarkable one, and will serve to direct attention anew to nuclein. Hitherto this agent has been studied solely with reference to its influence over the leucocytes, and this is the first time I have heard of its influencing the erythrocytes. That nuclein has powers that could be utilized in the treatment of disease has been evident from the time Vaughan's studies of it were made public. A faint objection was raised that, inasmuch as nuclein entered the stomach as a component of many foods, it could not act as a medicine in the relatively minute doses recommended. The same objection has been raised in regard to iron, lime and other remedies, but with all the sufficient argument holds good, that when administered in medicinal doses definite therapeutic results are obtained.

With nuclein, unassisted clinical observation is not enough, but we know by the microscope that when this remedy is injected subcutaneously there is an increase in the number and activity of the leucocytes. Now Dr. Ward enlightens us further; the red corpuscles are similarly affected by nuclein. Speaking from clinical observations alone, I have obtained good results from nuclein when administered in the mouth, the endeavor being made to have the solution absorbed by the buccal mucous membrane and not swallowed.

In the discussion that followed one speaker asserted that nuclein did not act so much as a natural constituent of the blood of the patient, but as a foreign and toxic agent, antagonizing the powers of the body. This brings up an interesting phase of the question: Tuberculin excites febrile reaction when injected; so do urea, creatin, creatinin, leucin, tyrosin, all the animal extractives as yet tested. So does the chemically allied thiosinamin; and very markedly so does crotalin, the rattlesnake venom introduced by Professor Mays. But is an agent that will arouse reaction necessarily an enemy? Take the case of a *foudroyant* attack of an eruptive or pernicious fever, where the patient is overwhelmed with the intensity of the attack and in imminent peril. Unless we can speedily arouse reaction the patient dies. So we administer glonoin, atropine and strychnine, in minute but quickly repeated doses, until the vitality is aroused and febrile reaction is established. But this is the most perfect type of the stimulant medication in therapeutics. So that the use of nuclein here can in no sense be looked upon as placing an additional burden on the patient's vitality, but as a means of arousing, developing and increasing that resistant power without which he must succumb to the malady.

Dr. Ward has not pushed his use of nuclein beyond the dose of a dram of the Abbott standard solution; and he is wise, for too large doses are followed by an enormous fall in leucocytosis, and if this is the case with the erythrocytes great harm might be done.

Most of those present expressed the conviction that Dr. Ward had opened an exceedingly interesting line of observation, and it is to be hoped that his treatment may be put to an extended test.

Intravenous injections are not usually made by the everyday practician, although there is no particular reason why he should not, but hypodermoclysis is easy enough. Few consumptives will object to any procedure that offers a ray of hope, and anything unusual is more apt to impress them favorably than the commonplace. Nuclein solution is not very irritating, and the dose may be mixed with an ounce of salt solution and injected with one of the hypodermic syringes employed in veterinary practice. The injection may be repeated as soon as all reaction has subsided.

1360 Fulton St., Chicago, Ill.

